# Health management of malnourished elderly in primary health care: a scoping review

**DOI:** 10.1186/s12875-022-01883-9

**Published:** 2022-11-03

**Authors:** Adriana Taveira, Bárbara Sousa, Patrício Costa, Ana Paula Macedo

**Affiliations:** 1 School of Medicine, Life and Health Sciences Research Institute (ICVS), University of Minho, Campus de Gualtar, 4710-057 Braga, Portugal; 2 ICVS/3B’s - PT Government Associate Laboratory, Braga/Guimarães, Portugal; 3 School of Psychology, University of Minho, Braga, Portugal; 4 Faculty of Psychology and Education Sciences of the University of Porto, Porto, Portugal; 5 Health Sciences Research Unit: Nursing (UICISA: E), Portugal/School of Nursing (ESE), Nursing School of Coimbra (ESEnfC), University of Minho, Campus de Gualtar, 4710-057 Braga, Portugal

**Keywords:** Malnutrition, Elderly, Process and outcome evaluation in health care, Scoping review

## Abstract

**Objectives:**

The aim of this study, as the first review directed at Primary Health Care, is to identify the screening practices and health outcomes related to the care provided by Family Health Teams to the malnourished elderly people/population.

**Methods:**

Following PRISMA and PICO strategies, searches were conducted in four electronic databases (PubMed, Web of Science, Scopus & EMBASE) on observational, qualitative, quantitative, or mixed studies, written in Portuguese, Spanish and English language, with participants of 65 years old or older at a community setting. The literature selected for this study ranges from the period 2011 to 2021; additional articles were included through reference lists.

**Results:**

From the 483 studies identified, 16 were considered eligible to use in this work. The Mini Nutritional Assessment (MNA) score appears as the main criteria of choice, however, a standardized practice in the health systems regarding the use of screening methods has not been demonstrated. Studies are more oriented towards the analysis of the relationship of mortality/morbidity and malnutrition than towards the relationship of the cost and quality of life and malnutrition of the elderly.

**Discussion:**

Malnutrition is one of the modifiable risk factors which contributes to the vulnerable condition of the elderly, with serious effects, especially when related to other comorbidities. Yet, several authors argue that the Primary Health Care intervention can minimize the negative impacts and improve the health outcomes.

The twenty-first century is witnessing one of the most relevant social changes, which Portugal is also related to. According to the National Statistical Institute [1], the Portuguese demographic pyramid reflects a marked population aging (the fifth-highest value and the third lowest value of the Renewal Index of Population at the European level). An aging population has a marked propensity for developing states of multi-morbidity, which projects functional disabilities with direct effects on the consumption of resources [2]. Although the health of the Portuguese population has improved in the recent decades, this has not been accompanied by policies that reflect the need for improvement and investment in the health care of the elderly, recognizing that the phenomenon may mirror an increase in the demand for health care services [3].

Rodrigues et al. [4] in their study, confirmed a high prevalence of multimorbidity (78.3%), increasing across age strata (72.8% for 65—69 years to 83.4% for ≥ 80 years). Hospitalization was reported by 25.8% of the individuals, concluding that the high prevalence of multimorbidity, associated with unhealthy lifestyles, of which diet stands out, is a predictor of vulnerability in the elderly, requiring dedicated intervention. This fact represents an enormous challenge to the health sector. There’s a high expectation on the Primary Health Care Services (PHCS) – seen as a privileged and first-line access route for anyone to the National Health Systems, given their mission. Thus, implementing and improving effective and rapid community intervention strategies that mobilize responses capable of satisfying the specific needs of this population is a demand for the health sector to address [5].

In Portugal, considering the overall effects of aging, the Ministry of Health [6] has approved the National Programme for the Health of the Elderly, recommending special attention to the elderly, and the intervention of health professionals in the case of elderly with malnutrition.

Nutrition is a key component in the health of the elderly population, capable of determining the quality of aging conditions. In this sense, the adequate nutritional status is the reflection of the balance between the elder's body food intake and the body's nutritional needs [7, 8]. The concept of malnutrition refers to a state resulting from a lack of nutrient absorption or intake that leads to changes in the body composition (decreased fat-free mass) and the body cell mass, with a consequent decrease in physical and mental function, associated with a more reserved clinical prognosis [9].

According to the National Programme for the Promotion of Healthy Eating [10], the population's inadequate eating habits are the fourth modifiable risk factor that most contributed to the loss of healthy life years (11.4% of the total number of deaths), especially malnutrition, particularly in the elderly population.

To be noted that malnutrition in the elderly is a current phenomenon often underdiagnosed that has not, yet, received its deserved attention. It is easily assumed as a natural and expected sign of aging, and therefore its early recognition becomes essential for appropriate and timely correction [11]. To highlight that across any of the different authors referenced for systematic reviews, the most common and cross-cutting thematic interest was the relationship between malnourished elderly and their hospitalization and institutionalization [12–17]. So, it is important to recognize the importance of re-conducting a new research study capable of validating the terms of analysis for the context of the potential PHC intervention. The study will be based on a methodology that structures a scoping review and simultaneously substantiates the following objectives:

I. To identify the screening instruments in the diagnosis of elderly´s malnutrition;
II. To identify the health outcomes (morbidity, mortality, functional capacity, and quality of life) associated with under-diagnosis and under-intervention of Family Health Teams (Doctors and Nurses) regarding the phenomenon of malnutrition in the elderly;

## Method

### Eligibility criteria

The selected studies complied with the following PICOS strategy. Only studies are written in English, Spanish and Portuguese languages were considered. We included data sources on observational, qualitative, quantitative, or mixed indexed studies, published between 2011–2021, addressed to the adults aged 65 years and over, describing the use of screening/intervention tools or the identification of health outcomes (morbidity, mortality, functional capacity and quality of life) associated with under-diagnosis and under-intervention of the Family Health Teams in face of the malnutrition phenomenon. We have published a protocol for a scoping review that can be retrieved from osf.io/sw3aj. https://doi.org/10.17605/OSF.IO/SW3AJ.s health.

### Information sources and search strategy

In July of 2021, the literature search was carried out by two reviewers (AT, BS) in PubMed, Web of Science, Scopus & EMBASE. The literature was selected from the year 2011 onwards. We opted to define this as the temporal interval to conduct our research, based on the systematic publication of reviews, in the recent years, regarding the theme of malnutrition in the elderly population [12–17]. Snowball citations were retrospectively and prospectively screened to ensure literature saturation. Review articles (i.e. systematic, scoping, narrative reviews), expert opinion excerpts, protocol articles, and trial registers were excluded. The search strategy is presented in Table 1.

**Table 1:** Search strategy

Search Criteria	Search Term
Population	(“elder” OR “elderly” OR “geriatric population”) NOT (“child*” OR “adolesce*” OR “adolescent*”
Intervention	(“screening tools” OR “treatments” OR “assessment”)
Outcomes	(“malnutrition” OR “undernutrition” OR “malnourished”) AND (“morbidity” OR “mortality” OR “functional capacity” OR “global Functions” OR “quality of life”)
Setting	(“primary care” OR “primary health care” OR “primary healthcare” OR “family practice” OR “family medicine” OR “general practice” OR “general practitioner” OR GP OR “family doctor” OR “family nurse”)

### Study selection

**Fig. 1:**
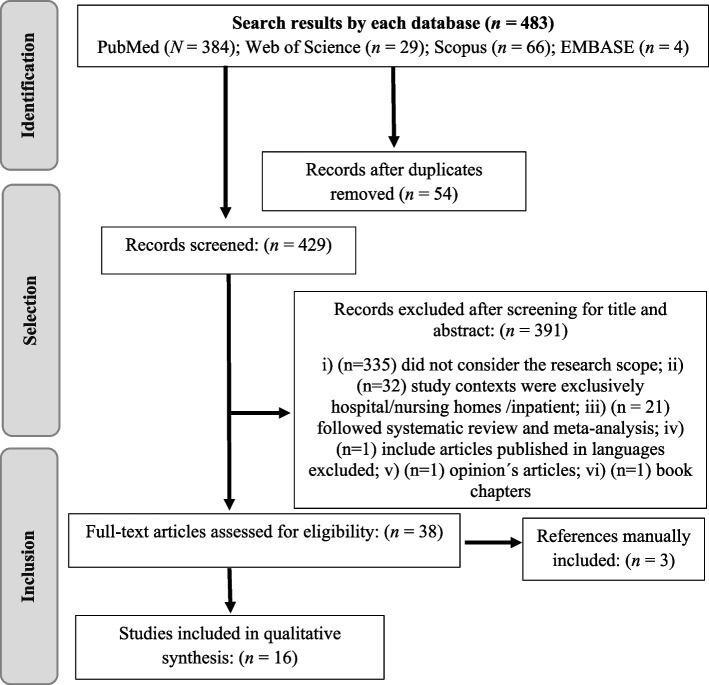
Adaptation of PRISMA flow chart

Studies founded by database analysis were exported to the Rayyan Management Software. Reviewers AT, AM selected the eligible studies for this review. An inclusion/exclusion algorithm was created to facilitate the data screening phases (three in total). In the first data screening phase, the duplicates identified by the software were reviewed and removed by AT, BS. In the second phase, two reviewers AT, AM independently identified assessed the titles and abstracts of the studies for inclusion. Each reviewer decided on the inclusion or exclusion of each paper based on the inclusion/exclusion criteria stated above. During the third phase, two reviewers AT, AM independently reviewed the papers approved during the second phase data review for inclusion. Possible disagreements were resolved by consensus. The reason for exclusion was identified for all excluded studies and a PRISMA flowchart was drawn to summarise the study and selection process (Fig. 1).

### Data extraction

The selected data was migrated to an Excel document format, and the information was organized according to studies that point out (1) the procedure (screening/intervention tools) that identified the malnutrition in the elderly; (2) and/or data from studies that identify health outcomes associated with under-diagnosis and under-intervention by Family Health Teams.

In addition, when possible, data were extracted on: 1) study characteristics (study design, country of origin, year of publication, and sample size); 2) participant characteristics (socio-economic and educational variables, clinical conditions, living arrangement, mean age, gender, and race); 3) screening instrument used in the diagnosis of malnutrition; 4) elderly´s health outcomes (morbidity, mortality, functional capacity and quality of life); 5) main screening instruments´ characteristics in the diagnosis of elderly´s malnutrition referred in the different studies; 6) main conclusions. The descriptions of the studies are presented in Tables 2, 3, and 4.

**Table 2:** Description of main sample characteristics, study design, and procedures

**Authors (Year)**	**Sample size (n)**	**Sample characteristics** (mean age, socio-economic and educational variables, clinical conditions, gender, living arrangement, and race)	**Design**	**Procedures (recruitment)**
Ahmed et al. [18]	*n* = 15 121 131 (801 272) malnourished	**Malnourished elderly** M = 75.4 years Social level = NR Educational level = NR Clinical conditions = 100% diabetes Gender = 57.63% women Living arrangement = NR Race = 74.93% whites	Observational retrospective study	Clinical diagnosis in the medical record (ICD-9 and 10)
		**Normo-nourished elderly** M = 71.3 years Social level = NR Educational level = NR Clinical conditions = 100% diabetes Gender = 52.79% women Living arrangement = NR Race = 79.24% whites		
Alhamadan et al. [19]	*n* = 2045 (427.4) risk/malnourished	**Risk/malnourished Elderly** M = 66.1 years Social level = 83.7% Not Employed Educational level = 27% Intermediate & Secondary Clinical conditions = 63.2% without comorbidities Gender = 55% women Living arrangement = 79.4% married Race = NR	Cross‐sectional descriptive study	MNA & BMI
Galiot et al. [20]	*n* = 57 (14.89) risk/malnourished	**Risk/malnourished Elderly** Age = range from 75 to 80 years (59.65%) Social level = NR Educational level = NR Clinical conditions = 63.2% without comorbidities Gender = 55% women Living arrangement = NR Race = NR	Cross‐sectional descriptive study	MNA
Geurden et al. [21]	*n* = 100 (29) risk/malnourished	**Risk/malnourished Elderly** M = 76.1 years Social level = NR Educational level = NR Clinical conditions = NR Gender = 86.2% women Living arrangement = NR Race = NR	Randomized cross sectional study design	MUST
Hegendörfer et al. [22]	*n* = 567 (69) were at risk of malnutrition based on MNA and (72) based on pre-albumin	**Malnourished elderly based on MNA** M = 85.6 years Social level = NR Educational level = NR Clinical conditions = NR Gender = 72.1% women Living arrangement = NR Race = NR	Observational prospective cohort study	MNA & prealbumin levels
		**Malnourished elderly based on pre-albumin levels** M = 95.9 years Social level = NR Educational level = NR Clinical conditions = NR Gender = 76.9% women Living arrangement = NR Race = NR		
Klemenc-Ketis et al. [23]	*n* = 1641 (216) risk malnourished	**Risk malnourished Elderly** Age = 60.4% (range from 20 to 99 years) Social level = NR Educational level = NR Clinical conditions = 43.6% Gender = 68.1% women Living arrangement = NR Race = NR	Cross-sectional observational study	MUST
Krishnamoorthy et al. [24]	*n* = 279 (50) risk/malnourished	**Risk/malnourished Elderly** Age = range from 60 to 70 years (62%) Social level = 96% unemployed Educational level = 88% no formal education Clinical conditions = NR Gender = 76% women Living arrangement = NR Race = NR	Cross‐sectional descriptive study	MNA
Mastronuzzi et al. [25]	*n* = 274 (99) risk/malnourished	**Risk/malnourished Elderly** M = 85 years Social level = NR Educational level = NR Clinical conditions = 23.3% dementia Gender = 63.63% women Living arrangement = NR Race = NR	Cross‐sectional descriptive study	MNA
Pedersen et al. [26]	*n* = 208 malnourished	**Intervention Group** M = 86 years Social level = NR Educational level = NR Clinical conditions = 35% comorbidities Gender = 59% women Living arrangement = 64.5% eat alone Race = NR	Randomized study	MNA
		**Control Group** M = 86.3 years Social level = NR Educational level = NR Clinical conditions = 32% comorbidities Gender = 55% women Living arrangement = 63% eat alone Race = NR		
Preston et al. [27]	*n* = 59 (37/59) malnourished based on ANSI (10/59) malnourished based on MNA (9/59) malnourished based on MUST (12/59) malnourished based on MST	**Malnourished elderly** Age = > = 80 years (60%) Social level = NR Educational level = NR Clinical conditions = 60% fracture risk Gender = 60% women Living arrangement = 70% living with another Race = NR	Mixed Study	MNA, ANSI, MUST & MST
Rodriguez-Tadeo et al. [28]	*n* = 760 (384) risk/malnourished	**Elderly** Age = range from 70 to 79 years (44.6%) Social level = NR Educational level = 41.8% Primary or lower Clinical conditions = 52.7% depression Gender = 63.63% women Living arrangement = 47.1% married Race = NR	Observational, descriptive, and cross-sectional study	MNA
Schilp et al. [29]	*n* = 146 malnourished	**Intervention Group** M = 80.6 years Social level = NR Educational level = NR Clinical conditions = NR Gender = 62.5% women Living arrangement = 43% living alone Race = NR	Randomized study	SNAQ 65 +
		**Control Group** M = 80.5 years Social level = NR Educational level = NR Clinical conditions = NR Gender = 66.2% women Living arrangement = 53% living alone Race = NR		
Shakersain et al. [30]	*n* = 3041 (802) risk/malnourished	**Risk/Malnourished Elderly** Age = 60–69 years Social level = NR Educational level = 42.6% High School Clinical conditions = 80.1% vascular disease Gender = 70.9% women Living arrangement = 65.2% living alone Race = NR	Observational prospective cohort study	MNA
Spirgienė et al. [31]	*n* = 169 (82) malnourished	**Malnourished Elderly** Age = range from 65 to 75 years (55%) Social level = NR Educational level = NR Clinical conditions = NR Gender = 63.9% women Living arrangement = 45% living with another person Race = NR	Cross‐sectional descriptive study	MNA
Vandewoude et al. [32]	*n* = 3299 (1389) risk/malnourished	**Risk/Malnourished Elderly** M = 82.7 years Social level = NR Educational level = NR Clinical conditions = 20% depression Gender = 69,5% women Living arrangement = NR Race = NR	Observational Cross-sectional study	MNA
Yang et al. [33]	*n* = 198 (124.7) risk/malnourished	**Risk/Malnourished Elderly** M = 78 years Social level = NR Educational level = 48.9% High School Clinical conditions = NR Gender = 84.9% women Living arrangement = 22.8% living with another person Race = 35.6% African American	Observational prospective cohort study	MNA

*ANSI* Australian Nutritional Screening Initiative, *BMI* Body Mass Index, *ICD* International Classifications of Desiese, *MNA* Mini Nutritional Assessment, *MUST* Malnutrition Universal Screening Tool, *MST* Malnutrition Screening Tool, *NR* Not Reported, *SNAQ 65* + Short Nutritional Assessment Questionnaire 65 +

**Table 3:** Description of main screening instruments´ characteristics in the diagnosis of elderly´s malnutrition referred in the different studies

**Screening Instruments**	**Nutritional profiles categories** (baseline)	**Application**	**Phsychometric Characterístics**
ANSI [27]	Not reported	Research/health professional assessment	Not reported
BMI [19]	Body mass index [weight in kg/(height in m)2 was used to classify participants as underweight (< 18.5 kg/m2), normal weight (18.5–24.9 kg/m2), overweight (25.0–29.9 kg/m2) and obese (≥ 30 kg/m2) Based on elder-BMI classification (aged ≥ 65 years), a BMI between 22–27 kg/m2 is considered a normal range. Therefore, the following cutoff points were also been used (< 22 kg/m2 under-weight, and > 27 kg/m2 for overweight)	Research/health professional assessment	Not reported
MNA [18–20, 22, 24–28, 30–33]	The tool consists of two parts: the screening and the assessment. The screening part includes BMI and questions regarding the decline of food intake, weight loss in the past three months, mobility, psychosocial stress or acute disease in the past three months, and neuropsychological problems The screening score ranges from 0 to 14 where 11–14 is considered normal nutritional status, 8–11 is a risk of malnutrition and 0–7 is malnutrition. If the screening score is ≤ 11 then the assessment part is implemented that consists of 12 items including mid-arm and calf circumferences and questions about feeding, self-assessment of nutrition and general health, taking more than three prescription drugs, independent living, and pressure sores/skin ulcers. The assessment score ranges from 0 to 16. Based on the scores of the screening and assessment parts of MNA, a malnutrition indicator score is calculated The answers can give three nutritional profile categories: 1) well-nourished (24–30 points); 2) risk of malnutrition (17–23.5 points; 3) undernourished (< 17 points)	Research/health professional assessment	It´s a reliable and easy-to-use nutritional assessment tool Has an adequate predictive validity and specificity of analysis concerning the frail elderly population Recommended by the European Society for Enteric and Parental Nutrition
MST [27]	Not reported	Research/health professional assessment	Not reported
MUST [21, 23, 27]	Assigns risk for malnutrition based on BMI (< 18.5 kg/m = 2; 18.5–20 = 1; > 20 = 0), unplanned weight loss in 3–6 months (< 5% = 0; 5–10% = 1; > 10% = 2), and acutely ill with no nutritional intake or likelihood of no intake for more than 5 days (= 2). A total score of 2 or more indicates high risk of malnutrition with treatment recommended. A score of 1 is interpreted as medium risk with recommended follow-up. A score of 0 indicates low risk with continual care as usual	Research/health professional assessment	MUST has face content, concurrent, and predictive validity. The tool is internally consistent and reliable, with a very good to excellent reproducibility when different observers assess the same patients (kappa values between 0.8 and 1.0) The community-dwelling population MUST predict rates of hospital admissions and General Practices visits. It also shows that appropriate nutritional intervention improves outcomes. MUST has been made user-friendly through extensive field testing by many professionals in different healthcare settings Recommended by the European Society for Enteric and Parental Nutrition, the American Society for Parenteral and Enteral Nutrition, and the National Institute for Health and Care Excellence
Prealbumin Levels [22]	The prealbumin was measured in fasting serum with the UniCel® DxC 800 Synchron. Based on prealbumin levels, the nutritional status categories were at risk of malnutrition (prealbumin < 20 mg/dl) and without risk of malnutrition (prealbumin ≥ 20 mg/dL)	Research/health professional assessment	Not reported
SNAQ 65 + [29]	The profile undernourished is considered if the mid-upper arm circumference is less than 25 cm and/or 4 kg or more self-reported unintentional weight loss within the past 6 months	Research/health professional assessment	Not reported

*ANSI* Australian Nutritional Screening Initiative, *BMI* Body Mass Index, *MNA* Mini Nutritional Assessment, *MUST* Malnutrition Universal Screening Tool, *MST* Malnutrition Screening Tool, *NR* Not Reported, *SNAQ 65* + Short Nutritional Assessment Questionnaire 65 +

**Table 4:** Description of elderly´s health outcomes (morbidity, mortality, functional capacity, and quality of life), and main conclusions of the eligible studies

**Authors (Year)**	**Elderly´s health outcomes** (morbidity, mortality, functional capacity, and quality of life)	**Main conclusions**
Ahmed et al. [18]	The risk of death for those with diabetes increased 69% in malnourished versus normal nutrients (*P* < 0.0001). Malnutrition increased the risk of death within reach of the common comorbid conditions, including ischaemic heart disease, chronic obstructive pulmonary disorder, stroke or transient ischemic attack, heart failure, chronic kidney disease, and acute myocardial infarction. In addition, the total annual expenditure for the malnourished beneficiaries were significantly higher than then for the normal-nutrient beneficiaries ($36 079 vs 20 787) (*P* < 0.0001)	Malnutrition is significant comorbidity affecting the survival and health care costs of the person with diabetes. There is a need to develop and implement evidence-based clinical decision pathways for appropriate screening, assessment, diagnosis, and treatment of malnourished patients, and to prevent malnutrition in normal-nutritious patients with diabetes
Alhamadan et al. [19]	There was a significant association between nutritional status and ADL. Only 15.2% were at risk of malnutrition or malnourishment among those assessed as fully functional. Among those rated as moderately functional or severely unfunctional, 55.3% and 73.9% were classified as at risk of malnutrition or malnourished, respectively	Assessing the nutritional status of the elderly identified a high prevalence of undernutrition and obesity. Such assessments should be routine practise in PHC
Galiot et al. [20]	The risk of malnutrition was positively related to social risk and chronic diseases. 3.6% of participants who had social problems, were at risk of malnutrition and malnutrition (1.8%). People suffering from more than six pathologies, also had a higher nutritional risk	The risk of undernutrition seems to be associated with a more disadvantaged social condition and comorbidities. The development of training programs in nutrition education and the use of simple tools to identify nutritional risk in primary health care could effectively reduce the prevalence of malnutrition, avoid negative health consequences and improve the quality of care. If a situation of nutritional risk is not detected and treated early, it can lead to malnutrition, a serious pathological situation with very negative consequences for the elderly´s health, not to mention the social and health costs that this situation entails
Geurden et al., [21]	Patients at risk of malnutrition were significantly sicker (*P* < 0.001), and revealed more eating problems such as difficulties with chewing or swallowing and loss of appetite (*P* < 0.001)	Belgian nurses providing care at home do not yet fully comply with international nutritional recommendations. Our survey of nurses revealed low awareness, low knowledge capacity, and poor communication between stakeholders. Systematic screening should be further developed and evaluated in this population at risk. Additional training in nutritional nursing care and screening methods for malnutrition is needed
Hegendörfer et al. [22]	Survival is statistically significantly lower for those with a risk of malnutrition based on either MNA (*P* < 0.001) or pre-albumin (*P* = 0.001). No significantly higher hospitalization is observed for those at risk of malnutrition based on MNA (*P* = 0.068) or pre-albumin (*P* = 0.058)	There is the need for further research on the assessment of nutritional status in community-dwelling, independent very old adults who would benefit from a combination of both anthropometric and/or dietary assessment and biomarkers such as pre-albumin. This is important in light of the growing global population of adults 80 years and older, the impact of malnutrition on their quality of life and risk of adverse outcomes, and the availability of potentially beneficial interventions
Klemenc-Ketis et al. [23]	There is a significant association of increased risk of malnutrition with age and BMI (*P* = 0.022); several chronic diseases (*P* = 0.001); a misperception of their current health status (*P* = 0.001); feeling lonely (*P* < 0.001); and increased pain intensity (*P* = 0.003)	A screening program in primary health care can help identify people at risk of malnutrition. In addition, appropriate nutritional support is suggested as it can help reverse or stop the trajectory of malnutrition and the negative outcomes associated with poor nutritional status. It should be noted that by limiting screening for malnutrition only to hospitalized and elderly patients, we are missing a large percentage of the population living at home, especially those who do not attend PHC
Krishnamoorthy et al. [24]	Not Reported	Nutritional dysfunctions are important health issues to be considered among the elderly population. Opportunistic screening may be useful at the PHC level. Strengthening primary health care to address and prevent this health issue through balanced dietary practices can improve their nutritional status, thereby improving their quality of life
Mastronuzzi et al. [25]	A significantly higher number of major events, including death, were observed in the undernourished group. In 20.4% of patients were identified as having a high risk of developing major complications in the future. The sensitivity of the MNA test in identifying these patients was 84%. Several major events were registered both in patients at risk of malnutrition and in malnourished ones (bone fractures – 20.5%; hospitalization – 30%; death – 2.8–16%)	The prevalence of malnutrition is high among the elderly in the family practice. The MNA allows for better identification of malnourished subjects than the BMI and effectively. The application of a simple, quick, and easy-to-fill screening tool such as the MNA makes it possible to identify better than BMI those older adults who are malnourished or at risk, which is also useful for quantifying the risk of future major events. Malnutrition is often underestimated as nutritional status is not routinely checked and reported in the patient's electronic file. The classification obtained by the MNA makes it possible to stratify patients and obtain information on the risk of complications, at least in older and frail subjects, and indirectly to estimate the burden of care
Pedersen et al. [26]	Early and integrated nutritional monitoring (Hosp-PHC) of the elderly by health teams in a home setting, prevents and improves the deterioration of activities of daily living and the independence of the individual when associated with malnutrition (in 96% of cases and statically significant *p* < 0.001). There is also a reduction in the length of hospital stay	The early identification of the risk of malnutrition can prevent a negative spiral of results for the elderly (functional deterioration, hospital readmissions, death)
Preston et al. [27]	50% of participants at nutritional risk were at risk of isolation. A large proportion of participants (79.9%) had multiple illnesses with almost 50% more than six prescribed medications. 80% of the nutritional risk group are considered frail. Frailty (*P* < 0.004) and prescribed medications (*P* < 0.042) reveal a statistically significant relationship with nutritional status	Screening practices with valid and reliable screening tools are imperative to ensure identification and management of older people at risk
Rodriguez-Tadeo et al. [28]	Functional impairment, cognitive impairment, and depression were 3.0, 1.5, and 2.9 times more likely in the presence of malnutrition or risk of malnutrition. Malnutrition correlates positively with depression, functional impairment to move, and living alone (*P* < 0.001)	PHC plays an important role in early detection, improved quality of life, and better prognosis for malnourished individuals
Schilp et al. [29]	No statistically significant differences were found between introduction of dietary treatment VS usual care in body weight change (mean difference 0.78 kg, 95% CI-0.26e1.82), QALYs (mean difference 0.001, 95% CI—0.04e0.04) and total costs (mean difference V1645, 95% CI -525e3547). The incremental cost-utility ratio (ICUR) for QALYs was not interpretable. The incremental cost-effectiveness ratio (ICER) for body weight gain was 2111. The probability of dietary treatment being cost-effective was 0.78 for a cost-effectiveness ratio of V5000 for body weight and 0.06 for a threshold ratio	Dietary treatment in older, undernourished, community-dwelling individuals was not cost-effective
Shakersain et al. [30]	The mean age at death of participants with malnutrition and risk of malnutrition was ~ 3 and 1.5 years shorter (CI—95%, *P* < 0.001) than that of participants with normal nutritional status, respectively, while malnutrition or risk of malnutrition together with abnormal biomarker levels (hemoglobin and albumin) was related to one year shorter survival	Malnutrition and the risk of malnutrition are significantly associated with shorter survival. Poor nutritional status in combination with abnormalities in biomarkers is associated with even shorter survival
Spirgienė et al. [31]	The risk of/malnutrition was associated with chronic (*P* < 0.004) and intermittent pain (*P* < 0.001), chewing difficulties (*P* < 0.001), swallowing disorders (*P* < 0.001), dental problems (*P* < 0.001), and medication use (*P* < 0.001). The risk of malnutrition and undernutrition was related to depression (*P* = 0.001) and Alzheimer's disease or other dementia (*P* < 0.001), but had no statistically significant relationship with cancer (*P* = 0.120) or diabetes mellitus (*P* = 0.065)	Educating community elders about healthy nutrition and providing them with specific updated guidelines to follow over the long term contributed to favorable changes. The findings infer that community nurses' efforts to ensure better health outcomes for the elderly, using minimal financial and human resources, appeared to be effective in improving elderly people's nutrition knowledge and practices on nutrition
Vandewoude et al. [32]	Of all undernourished individuals, 49% were diagnosed by PHC and 13% of the undernourished recognized themselves as such. Mobility (climbing stairs and walking) and ADL dependence (Belgian KATZ score) were impaired in older people with (risk of) malnutrition compared to individuals with normal nutritional status (*P* < 0.001)	Under-diagnosis of malnutrition is problematic, because the associated loss of mobility and independence may accelerate the transformation of frailty into disability in older people
Yang et al. [33]	Participants who were malnourished or at risk of malnutrition were more likely to experience sequential under-hospitalization (*P* = 0.040), emergency room visits (*P* = 0.047), use of home health aides (*P* = 0.027), and mortality (*P* = 0.031)	Malnourished people or at risk of malnutrition, are more likely to use greater amounts of health care resources subsequently and experience mortality. Nutritional interventions aimed at addressing undernutrition in this vulnerable population can improve health outcomes and decrease health service utilization

*ADL* Activities Day Living, *BMI* Body Mass Index, *KATZ* Index Independence Activities Day Living, *MNA* Mini Nutritional Assessment, *QALYs* Quality Adjusted Life Years

### Data synthesis

The included articles were submitted to a qualitative synthesis. The main results were organized into different categories in a discrete and non-overlapping manner. In each category, the results were summarised, highlighting their meaning.

## Results

Initially, 483 studies were identified through an electronic database search. After removing duplicates (*n* = 54), the titles and abstracts were screened, and 391 studies were excluded. The main reasons for the exclusion of the studies were: i) did not consider the research scope (*n* = 335); ii) study contexts were exclusively hospital/nursing homes /inpatient (*n* = 32); iii) followed systematic review and meta-analysis methodology (*n* = 21); iv) include articles published in excluded languages (*n* = 1); v) opinion articles (*n* = 1); vi) book chapters (*n* = 1). Of the 38 full-text articles assessed for eligibility, 13 met the inclusion criteria, and the other 25 were excluded because they presented unclear data regarding the report of our main objectives. References of these studies were manually analyzed, resulting in 3 additional studies. In total, 16 studies were identified for this scoping review (Fig. 1).

### Characteristics of included studies

#### Country of origin, year of publication, and sample sizes

The last ten years (between 2011 and 2021) evoke research interest in the topic across a broad geographical context, most notably in Belgium (18.75%) [21, 22, 32] and the USA (12.5%) [18, 33], followed by, Saudi Arabia [19], Spain [20], Slovenia [23], India [24], Italy [25], Denmark [26], Australia [27], Mexico [28], the Netherlands [29], Sweden [30] and Lithuania (respectively with 0.06%) [31]. Sample sizes of the included studies ranged from a minimum of *n* = 57 [20] to, a maximum of *n* = 15 121 31 [18] (Table 2).

#### Sample characteristics

The sample characteristics of the included studies reveal that the age of the participants ranged between 60 [23, 24, 30] and 99 years [23]. Most of the malnourished elderly were female [18–24, 26–29, 31], lived with another person [19, 27, 28, 31], and had associated comorbidities [18, 23, 24, 26–28, 30, 32, 33]. It should be noted that given the other terms that we previously established in the analysis of the sample characteristics, only two studies considered the assessment of the socio-economic level of the participants [19, 24] five studies reveal the educational level [19, 24, 28, 30, 33] and two consider race [18, 33] (Table 2).

#### Design

The research design was mostly observational, and cross-sectional descriptive studies (75%) [18–20, 22–25, 28, 30–33], followed by quantitative studies (18.7%) [21, 26, 29], and mixed (0.06%) [27]. The dissemination of the articles was essentially from the health area (56.2%) [18, 21, 22, 24, 27, 29, 31–33], and from the nutrition scope (43.8%) [19, 20, 23, 25, 26, 28, 30] (Table 2).

#### Procedures

Fifteen samples of malnourished elderly were obtained based on validated screening instruments [19–33] and only one study [18] based on the clinical diagnosis made by the physician, reported in the elderly patient's clinical file (Table 2).

#### Measures/malnutrion screening instruments

Twelve studies used the Mini Nutritional Assessment (MNA) to diagnose malnutrition in the elderly [19, 20, 22, 24–28, 30–33]. To highlight that nine of those twelve studies used MNA exclusively for the sample selection [20, 24–26, 28, 30–33]. Hegendörfer et al. [22] used it combined with pre-albumin levels, and Preston et al. [27] used it in association with three other screening instruments (namely, Australian Nutritional Screening Initiative – ANSI; Malnutrition Universal Screening Tool – MUST and Malnutrition Screening Tool—MST). Schilp et al. [29] appealed to Short Nutritional Assessment Questionnaire 65 + (SNAQ 65 +). Galiot et al. [20] and Klemenc-Ketis et al. [23] just used MUST. Ahmed et al. [18] does not consider the screening instrument. The study selected malnourished participants based on the clinical diagnosis in the medical record (ICD-9 and 10). The description of main screening instruments´ characteristics in the diagnosis of elderly´s malnutrition reveals that fourteen studies [19–26, 28–33], in their methods sections, explained the nutritional profiles categories (baseline) associated with the choice of their instruments, except for Preston et al. [27] that did not reported any information. Also, only three studies [21, 28, 33] have tried to sustain their instrument's options considering its psychometric characterístics and the recommendation of the Nutrition Group (the European Society for Enteric and Parental Nutrition, the American Society for Parenteral and Enteral Nutrition, and the National Institute for Health and Care Excellence). In all studies, the instrument´s application was assumed by the research/health professional assessment (Tables 2 and 3).

#### Elderly´s health outcomes

Concerning health outcomes in the elderly at risk of malnutrition, the analysis of the articles reveals a greater interest in studying the phenomenon associated with morbidity (37.5%) [20, 23, 25, 27, 28, 31], followed by mortality (31.25%) [18, 22, 25, 30, 33], activities day living (ADL) (25%) [19, 26, 28, 32], hospitalization (25%) [22, 25, 26, 33], social risk (18.75%) [20, 27, 28], and lastly, health costs and functional capacity (12.5%). In terms of the results, the study of Krishnamoorthy et al. [24] only carried out the characterization of the sample (highlighting the prevalence of malnutrition in the elderly, without its correlation with the variables under analysis). However, we opted for its inclusion considering the relationship between the diagnosis phase and the value of PHC intervention. Only Schilp et al. [29] have established the relationship between malnutrition and the quality of life.

Moreover, based on the description of the elderly´s health outcomes, and main conclusions of the eligible studies (Table 4), it allows us to state that the effects of malnutrition in the elderly, in terms of associated health outcomes, tend to be severe, especially when related to other comorbidities. Ahmed et al. [18] concluded that mortality in an elderly person with diabetes and malnutrition increases by 69%, including ischaemic heart disease; chronic obstructive pulmonary disease; stroke, or transient ischaemic stroke; chronic renal failure, and acute myocardial infarction. In addition, the total annual expenditure on health care for the undernourished individual was significantly higher. However at this point, to Schilp et al. [29], no statistically significant differences were found between the introduction of dietary treatment VS usual care in total costs.

Shakersain et al. [30], found that malnutrition and malnutrition risk was significantly associated with all-cause mortality and shortened survival by 3 and 1.5 years respectively. They also found that being elderly, living alone, and institutionalized directly correlated with poor nutritional status. However, the pure effect of malnutrition on mortality may not be perceived. In the present study, the relationship between poor nutritional status and mortality appears to be independent of chronic diseases suggesting that subclinical changes may play a role in the association between poor nutritional status and mortality. But then, Yang et al. [33], supports the previous analyses by robustly stating by their study that malnutrition in the elderly is assumed to be a risk factor for increased health service utilization and mortality [22, 25, 26].

The risk of malnutrition is identically related to lower physical and cognitive performance, greater functional disability (in terms of autonomy in ADL), and even entails an increased risk of depression and isolation [19–22, 27, 28, 31, 32]. In this connection, Yang et al. [33], and Mastronuzzi et al. [25], affirm that malnourished participants were more likely to experience sequential under-hospitalization, emergency room visits, use of home health aides, and mortality. This confirms the idea of Rodrigues et al. [28], when verifying that the high prevalence of multimorbidity, associated with unhealthy lifestyles, of which diet and its effects stand out, is a predictor of vulnerability, of increased hospitalization in the elderly.

Schilp et al. [29] assume that the improvement in quality of life occurs after body weight gain, which confirms the hypothesis of an association between body weight change and quality of life. Their study found that one-fifth of the participants were determined to have nutritional risk, identifying as promoting factors: poverty, poor oral health, medication use (*P* = 0.042), and social isolation. Inherent to this perspective, for Preston et al. [27], frailty itself is statistically significant in increasing their nutritional risk (*P* = 0.004), which is why it is possible to argue that the relationship between both is reciprocal and dependent.

## Discussion

### Summary of evidence

The study of elderly´s malnutrition in the community setting has been largely examined in the literature, however, no scoping review is currently available regarding the health processes and outcomes in PHC. In this context, it should be noted that PHC represents a key vector for intervention in promoting healthy eating habits and the prevention of malnutrition [34]. A commitment to differentiated intervention assumes a guide by the synergy of efforts of multidisciplinary teams. This means that the absence of a scoping review about this topic reveals to be a major gap in the literature.

To fill this gap, the present review was conducted (including 16 articles for analysis) with the purpose of synthesizing the data regarding the following objectives: i) to identify the screening instruments in the diagnosis of elderly´s malnutrition; ii) to identify the health outcomes (morbidity, mortality, functional capacity, and quality of life) associated with under-diagnosis and under-intervention of FHT regarding the phenomenon of malnutrition in the elderly.

Reading the articles allows us to state that the study of malnutrition´s association and morbidity had a wide range of interest (37.5%), compared to the other health outcomes (mortality, ADL, hospitalization, social risk, health systems costs, and quality of life, those latter, the least studied). However, overall, and as suggested by previous corroborating [7–9], it gets reinforced that the presence of malnutrition tends to accelerate the transformation of frailty into disability and worsen the elderly´s health (prognosis of other diseases, ADL, social risk, and quality of life), increasing the service utilization, but with some reservations for the mortality correlation. In some articles, this aspect may not be perceived, but in others, it is assumed to be a risk factor for increased mortality. At this point, we keep having some doubts about the pure effect of malnutrition on mortality.

Concerning the assessment´s process of risk/ malnutrition in the elderly, from the perspective of diagnosis and subsequent health intervention, it was observed that, although different screening instruments are available, the MNA, recommended by the European Society for Enteric and Parental Nutrition, showed a higher criterion of choice by researchers (75%). Still, two studies have justified it by its adequate predictive validity and specificity of analysis regarding the frail elderly population, (12,5%).

On the other hand, we could see that being elderly, living alone, or institutionalized is directly correlated with poor nutritional status, which sustains some properly, and special health policies recommendations [6], in attempting to reconduct the clinical good practices to the elderly being cared for. Whereas this fact, it seems to have results that confirm the hypothesis of an association between body weight gain and health outcomes improvement. Nevertheless, underlining some studies' recommendations, the dietary treatment should probably be prolonged (at least one year) and thus it will influence the individual's homeostasis positively.

It seems important to highlight that the combination of different results indicates that health professionals' timely and continuous action, especially in PHC, can positively influence the functional outcomes and dietary patterns of the elderly.

### Implications for the practice

In the qualitative synthesis analysis of different studies, we also identified some problems to the FHT/health System concerning their interventions profile related to the elderly´s malnutrition and some potential solutions to those obstacles, which we consider relevant to summarize below.

In PHC practices, health professionals do not: a) demonstrate standardized practices in the use of screening instruments [11, 25]; b) underestimate malnutrition in the elderly (assuming it a natural part of aging) [25, 32]; c) elderly´s malnutrition is not routinely checked and reported in the patient's electronic file [25]; d) revealed low awareness, low knowledge capacity, and poor communication between stakeholders [21]. However, some recommendations are addressed: a) to use scientifically validated assessment tools (it standardize clinical diagnoses of malnutrition, ensure identification and the early intervention) [23, 24, 26, 27]; b) to use a simple, quick, and easy-to-fill screening tool such as the MNA makes it possible to identify better than BMI [25]; c) encouraged to adopt a routine of good practices intrinsic to the overall assessment/intervention of the elderly, always from a perspective of care integration (Hospital – PHC) [19, 23]; d) to develop training programs in nutrition education and the use of simple tools to identify nutritional risk in PHC [20, 21]; e) to improve the communication between stakeholders; f) to strength PHC to address and prevent this health issue through balanced dietary practices [24]; and, g) to educate community elders about healthy nutrition and provide them with specific updated guidelines [31].

### Strengths and limitations

To the best of our knowledge, this is the first scoping review that synthesizes the range of knowledge available on PHC processes and health outcomes associated with malnutrition in elderly people. This reveals the greatest strength of the current study. The inclusion of peer-reviewed scientific articles published in English, Portuguese and Spanish, with a timeframe that includes the last 10 years of research on the current and growing phenomenon of vulnerability in the elderly, is another possible strength of the scoping review. However, we know that it may have limited the analysis by exclusion.

Given the interest of the current systematic reviews, it contrasts the need for scientific investment in this area of intervention, the PHC. Efforts were made to capture all relevant articles, assumed by the decision and interest to consult the references of eligible studies; however, articles could be overlooked. Including studies with different sampling methods reveals another possible strength of scoping review (as it is advisable to present papers that support and reinforce the results, overcoming the limitation of those with a less representative number of participants). However, the same condition may represent a possible associated limitation. Studies with different sampling representativity are included and compared, limiting the extrapolation of results.

## Conclusion

This scoping review has fulfilled the objectives settled, identifying important malnutrition screening instruments (in terms of frequency of use, baseline, and psychometric characteristics), and the impactful health outcomes associated with under-diagnosis and under-intervention of FHT in PHC (revealing understudied content areas). At this point, it is important to say that, in this scoping review, reporting the main screening instruments´ characteristics in the diagnosis of elderly´s malnutrition referred among the different studies (Table 3), with various methodologies designs may be useful for future research in the area, providing information for assessing their applicability, recommendation, and validity.

Regarding the phenomenon of malnutrition in the elderly, and among all articles analyzed, the study also summarises some of the problems that the FHT/health systems are facing, which should be considered and valued in the particular PHC setting. The scientific process is expected to support the definition of quality interventions/clinical governance that facilitate and promote decision-making in FHT.

The current socio-economic situation, aggravated by a pandemic, has led countries, like Portugal, to an unprecedented economic crisis. Associated with this macro context, an increase in the phenomenon of malnutrition is foreseeable, given that people may lose income and see their purchasing power reduced, aspects that will influence the acquisition of foodstuffs. Thus, the possible consequences arising from this new reality should be a call to researchers to invest time in analysis and intervention on the phenomenon of malnutrition in the elderly.

## Data Availability

All data analysed for and in this study is included in this published article.
